# Lipid Profile Changes Induced by Chronic Administration of Anabolic Androgenic Steroids and Taurine in Rats

**DOI:** 10.3390/medicina55090540

**Published:** 2019-08-27

**Authors:** A.E. Rosca, Camelia Sorina Stancu, Corin Badiu, Bogdan Ovidiu Popescu, Radu Mirica, Constantin Căruntu, Serban Gologan, Suzana Elena Voiculescu, Ana-Maria Zagrean

**Affiliations:** 1 Department of Functional Sciences, Division of Physiology and Neuroscience, “Carol Davila” University of Medicine and Pharmacy, 050470 Bucharest, Romania; 2 “Victor Babeş” National Institute of Research-Development in the Pathology Domain, 050096 Bucharest, Romania; 3 Department of Lipoproteins and Atherosclerosis, “Nicolae Simionescu” Institute of Cellular Biology and Pathology of the Romanian Academy, 030167 Bucharest, Romania; 4 Department of Endocrinology, “C. I. Parhon” National Institute of Endocrinology, “Carol Davila” University of Medicine and Pharmacy, 050470 Bucharest, Romania; 5 Department of Neurology, “Colentina” Clinical Hospital, “Carol Davila” University of Medicine and Pharmacy, 020350 Bucharest, Romania; 6 Department of Dermatology, “Prof. N.C. Paulescu” National Institute of Diabetes, Nutrition and Metabolic Diseases, 030167 Bucharest, Romania; 7 Department of Gastroenterology, “Elias” Emergency Hospital, “Carol Davila” University of Medicine and Pharmacy, 011461 Bucharest, Romania

**Keywords:** anabolic androgenic steroids, AAS, nandrolone decanoate, DECA, taurine, lipids, triglycerides, HDL-C, cholesterol, lipoprotein

## Abstract

*Background and Objectives*: Anabolic androgenic steroids (AAS), used as a therapy in various diseases and abused in sports, are atherogenic in supraphysiological administration, altering the plasma lipid profile. Taurine, a conditionally-essential amino acid often used in dietary supplements, was acknowledged to delay the onset and progression of atherogenesis, and to mitigate hyperlipidemia. The aim of the present study was to verify if taurine could prevent the alterations induced by concomitant chronic administration of high doses of AAS nandrolone decanoate (DECA) in rats. *Materials and Methods*: Thirty-two male Wistar rats, assigned to 4 equal groups, were treated for 12 weeks either with DECA (A group), taurine (T group), both DECA and taurine (AT group) or vehicle (C group). Plasma triglycerides (TG), total cholesterol (TC), low-density lipoprotein cholesterol (LDL-C), high-density lipoprotein cholesterol (HDL-C), hepatic triglycerides (TGh) and liver non-esterified fatty acids (NEFA) were then determined. *Results*: DECA elevated TG level in A group vs. control (*p* = 0.01), an increase prevented by taurine association in AT group (*p* = 0.04). DECA decreased HDL-C in A group vs. control (*p* = 0.02), while taurine tended to increase it in AT group. DECA decreased TGh (*p* = 0.02) in A group vs. control. Taurine decreased TGh in T (*p* = 0.004) and AT (*p* < 0.001) groups vs. control and tended to lower NEFA (*p* = 0.08) in AT group vs. A group. Neither DECA, nor taurine influenced TC and LDL-C levels. *Conclusions*: Taurine partially prevented the occurrence of DECA negative effects on lipid profile, suggesting a therapeutic potential in several conditions associated with chronic high levels of plasma androgens, such as endocrine disorders or AAS-abuse.

## 1. Introduction

Nandrolone decanoate (DECA) is one of the anabolic androgenic steroids (AAS) developed for therapeutic purposes in various disorders, such as deficient or absent male gonadal function, bone marrow failure syndrome, catabolic states, or breast cancer [1,2]. AAS misuse has become a major public health challenge following its continuous increase in sporting environments [1,3]. Accumulating evidence from a large number of human and animal studies has shown that high doses of AAS exert a detrimental influence on the cardiovascular system, inducing hypertension, dyslipidemia, impaired myocardial function or coagulation abnormalities, sometimes with severe end-points (thromboembolic events, arrhythmias, or sudden death) [4,5,6,7]. Numerous studies have pointed out the atherogenic effect of AAS in supraphysiological administration, leading to severe consequences: increased oxidative stress and inflammation of intima, endothelial dysfunction, alteration of serum low-density lipoprotein cholesterol (LDL-C) and high-density lipoprotein cholesterol (HDL-C), abnormal level of plasma lipoprotein (a) and homocysteine [4,8]. However, data regarding the effects of widely abused DECA on plasma lipid profile are still equivocal [8]. Also, there is a small amount of available information regarding the effect of DECA on hepatic triglyceride levels. Therefore, further research in this field would be of certain interest.

Taurine is a “semi-essential”, ubiquitous sulfur-containing amino acid in mammalian tissues, with important physiological functions such as: bile salts synthesis, regulation of intracellular Ca^2+^ concentration, anti-oxidative, osmoregulating, anti-inflammatory, anti-apoptotic, and cyto-protective actions [9,10,11]. Multiple human and animal studies have highlighted the role of taurine in cardiovascular disease prevention through various beneficial actions, such as cardioprotection, blood pressure regulation, hemostasis modulation, delay of atherogenesis, obesity prevention, protection against diabetes mellitus, and its complications [12,13,14,15]. Taurine has antiatherogenic properties that are provided through several mechanisms: it lowers cholesterol and homocysteine levels, stimulates bile salts synthesis, alleviates endothelial dysfunction, inhibits proatherogenic functions of monocytes, inhibits cell proliferation and prevents blood vessel remodeling [10,14,16]. The aim of the present study was to verify if taurine could prevent the alterations induced by chronic high doses DECA administration in rats. To our knowledge, there are no other studies assessing the influence of the two drugs on the lipid profile, when simultaneously administered.

## 2. Materials and Methods

### 2.1. Animals, Chemicals and Experimental Design

Thirty-two male Wistar rats, aged 14 weeks at the beginning of the study and weighting 323 ± 27 g (mean ± SD) were used. Animals were housed in individual cages floored with wood shavings, in a room with constant temperature (23 °C) and 12-h light-dark cycle (lights on at 07:00 h), with free access to rat chow and water. Depending on the treatments administered, rats were assigned to 4 groups (*n* = 8/group). The animals received a treatment for 12 weeks as follows: (1) A group received DECA (NANDROLONE DECANOATE^®^, Norma Hellas Pharmaceutical Industry, Athens, Greece) in a weekly intragluteal injection of 10 mg/kg body weight, diluted in a vehicle consisting in sesame oil with benzyl alcohol (90:10, *v/v*, 15 mg/mL); (2) T group received taurine (Merck KGaA, Darmstadt, Germany) as a supplement in drinking water (2%, 159.8 mmol/L), and a weekly intramuscular injection of vehicle; (3) AT group received both drugs (DECA and taurine), in similar doses, as used in the aforementioned groups; (4) C group was subjected to a weekly intramuscular vehicle injection. DECA dose was chosen according to the previous studies in rats and was comparable to that reported as being frequently used by abuser athletes, 8 mg/kg/week or approximately 600 mg/week [17].

**Figure 1:**
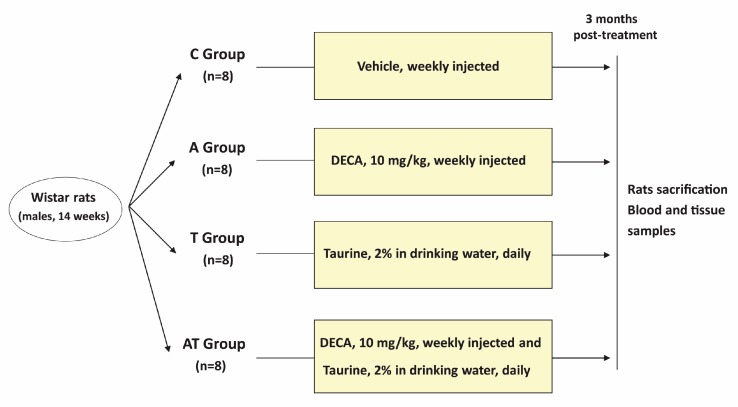
Graphical representation of the study design. The figure illustrates the main steps of the experiment: administration of vehicle in control group (C Group), administration of nandrolone decanoate (DECA) in androgen group (A Group), administration of taurine in taurine group (T Group), and co-administration of the two drugs in the mixed treated group (AT Group) for a period of 3 months; n - represents the number of rats in each group of the study.

After 12 weeks, rats were sacrificed by cardiac puncture, performed under ether induced and maintained anesthesia. Blood samples were collected into plastic tubes (3.8% sodium citrate, 1:9 citrate:blood, *v/v*), kept at 4 °C and immediately used for the lipid profile assessment. Liver was harvested, weighed and maintained at −70 °C until lipids measurements. The study design is summarized in Figure 1.

All animal procedures were approved by local ethics committee for animal research of “Nicolae Simionescu” Institute of Cellular Biology and Pathology, Bucharest, Romania (approval number 691, from 21 May 2012) and were carried out in accordance with the guiding principles for biomedical research involving animals as stated by the European Communities Council Directive 86/609/EEC.

### 2.2. Plasma Lipid Profile Analysis

Total cholesterol (TC), triglycerides (TG), LDL-C and HDL-C plasma levels were measured using commercially available kits from DIALAB GmbH, Vienna Neudorf, Austria (Cholesterol CHOD-PAP, Triglycerides GPO-PAP, Cholesterol LDL Direct and Cholesterol HDL Prec.) according to the manufacturer’s instructions.

### 2.3. Liver Lipids Measurement

Liver tissue samples (~300 mg frozen tissue) were homogenized in 500 µL phosphate buffer saline containing sodium dodecyl sulfate 1% on ice, using a DIAX 900 homogenizer (Heidolph Instruments GmbH & Co.KG, Schwabach, Germany) and centrifuged for 10 min at 10.000× *g* and 4 °C. The supernatant was collected and used for hepatic triglycerides (TGh) assay using the above-mentioned kit. The non-esterified hepatic fatty acids (NEFA) content of tissue homogenates was determined using a kit from Wako Chemicals GmbH, Neuss, Germany (NEFA-HR R1, NEFA-HR R2 and NEFA C standard). All parameters were expressed relative to tissue protein level measured by bicinchoninic acid (BCA) method (according to Sigma-Aldrich GmbH protocol, Steinheim, Germany) and using bovine serum albumin (BSA) as standard.

### 2.4. Statistical Analysis

Statistical analysis was performed using SPSS v.20.0 software for Windows (SPSS Inc. Chicago, IL, USA). Since all variables had a Gaussian distribution (Kolmogorov-Smirnov homogeneity test), one-way analysis of variance (ANOVA) was used for multiple comparisons between groups, followed by Tukey post-hoc test. Results are reported as mean values ± standard deviation (SD) and differences were considered significant when *p*-value < 0.05.

## 3. Results

**Figure 2:**
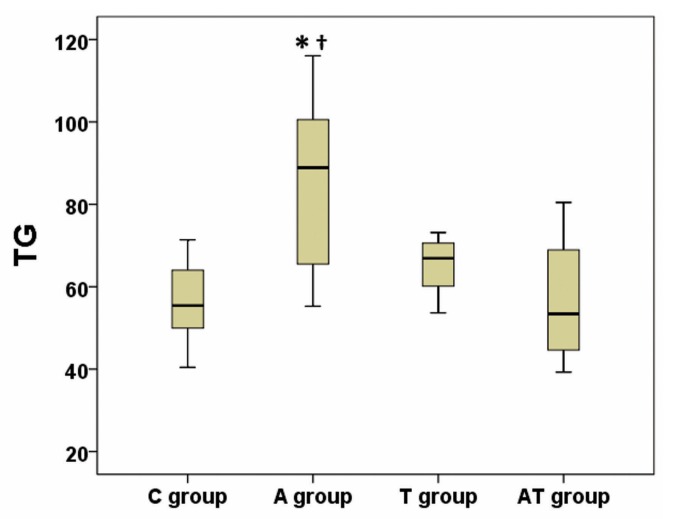
Influence of nandrolone decanoate, taurine and their combination on plasma triglycerides (TG) in rats. There was a significant increase of TG in the nandrolone decanoate treated group (A group) versus control group (C group) and also vs mixed treated group (AT group); T group represents the taurine treated group; *n* = 8 for each group of rats; TG is expressed in mg/dL; * represents *p* = 0.01 vs C group; † indicates *p* = 0.04 vs AT group.

**Figure 3:**
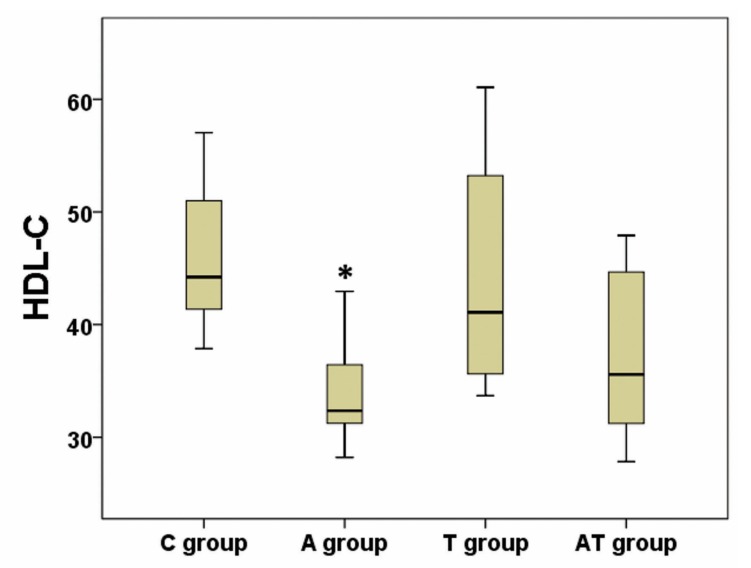
Influence of nandrolone decanoate, taurine and their combination on high density lipoprotein cholesterol (HDL-C). There was a significant decrease of HDL-C in the nandrolone decanoate treated group (A group) vs control group (C group); T group represents the taurine treated group, and AT group represents the mixed treated group; HDL-C is expressed in mg/dL; * represents *p* = 0.02 vs C group.

**Figure 4:**
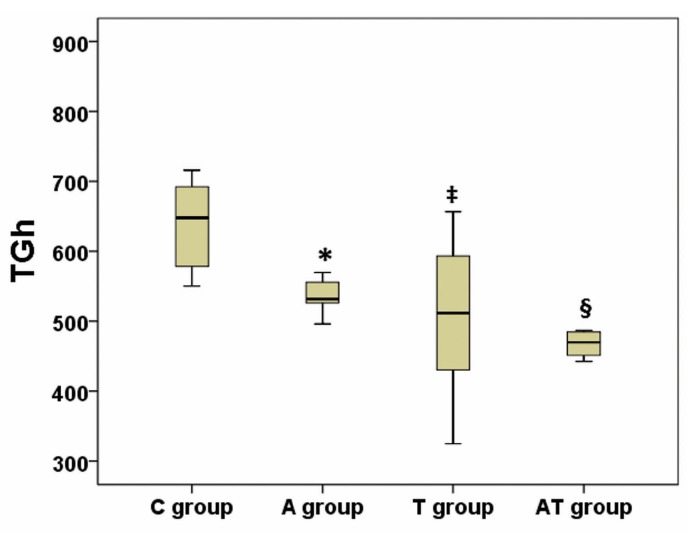
Influence of nandrolone decanoate, taurine and their combination on liver triglycerides (TGh). There was a significant decrease of TGh in the nandrolone decanoate treated group (A group), taurine treated group (T group) and mixed treated group (AT group) vs control group (C group); TGh is expressed in µg/mg tissue protein; * represents *p* = 0.02 vs C group; ‡ indicates *p* = 0.004 vs C group; § represents *p* < 0.001 vs C group.

**Figure 5:**
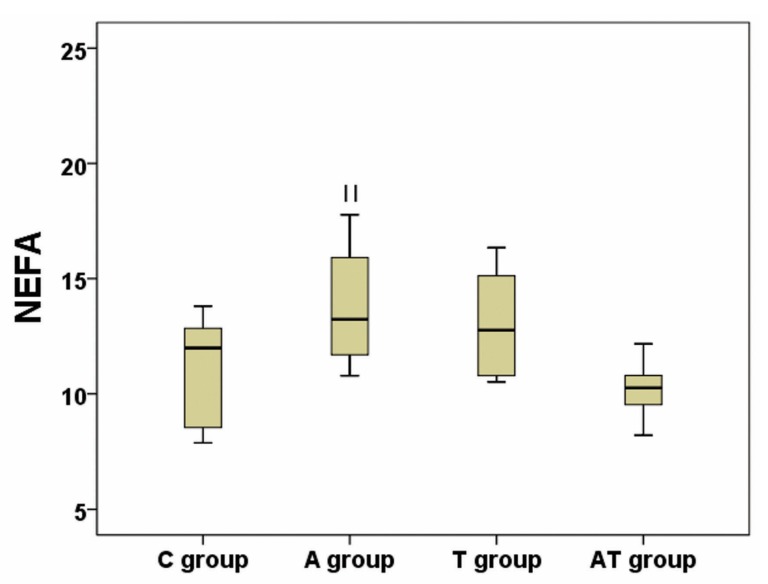
Influence of nandrolone decanoate, taurine and their combination on liver non-esterified fatty acids (NEFA). There was a significant increase of NEFA in the nandrolone decanoate treated group (A group) vs control group (C group); T group represents the taurine treated group, and AT group represents the mixed treated group; NEFA is expressed in µmol/mg tissue protein; || represents *p* = 0.08 vs AT group.

Values for TG, TC, LDL-C, HDL-C, TGh and NEFA are presented in Table 1. Using the analysis of variance, we have obtained a significant difference between groups regarding plasma TG, HDL-C and TGh (*p* ANOVA = 0.01; 0.01; <0.001 respectively), whereas a high tendency to statistical significance was achieved (*p* ANOVA = 0.06) regarding NEFA (Table 1). Post-hoc analysis identified an increase of TG in A group compared to C group (*p* = 0.01) and AT group (*p* = 0.04), while there were no statistical differences between AT and C groups (*p* = 0.97), or between T and C groups (*p* = 0.86) (Figure 2). Post-hoc analysis of HDL-C revealed a decrease in A versus C group (*p* = 0.02) (Figure 3). No significance was registered following comparisons of the four groups concerning TC or LDL-C. The ratio between liver weight and body weight (LW/BW) was not significantly modified (*p* ANOVA = 0.21) between groups at the end of the experiment (Table 1). Post-hoc TGh analysis revealed a decrease in A group (*p* = 0.02), T group (*p* = 0.004) and AT group (*p* < 0.001) as compared to C group (Figure 4), while NEFA evaluation identified only a tendency to increase in A group as compared to AT group, but without reaching statistical significance (*p* = 0.08) (Figure 5).

## 4. Discussion

Atherosclerosis-related cardiovascular disease remains the leading cause of morbidity and mortality worldwide [18]. Dyslipidemia by itself or in association with other cardiovascular risk factors is linked to atherosclerosis development [18]. Altered levels and functions of plasma lipoproteins have often been observed in several conditions associating increased concentrations of circulating androgens, such as: endocrine diseases (polycystic ovary syndrome, adrenal virilizing syndrome, enzyme defects of testosterone production etc) [19,20,21], AAS administration for different clinical conditions (patients with HIV, hemodialyzed patients etc.) [22,23], or non-medical use of AAS to enhance performance or appearance in athletes or amateur bodybuilders [5]. Even if the effects of AAS on lipoproteins are probably their most studied cardiovascular actions, and even if exogenous androgens were proven to induce major lipid profile alterations, data regarding the influence of DECA, a 17-β-esterified AAS on lipids are still equivocal [8].

In our study, supraphysiological administration of DECA has induced a significant increase of TG and a decrease of HDL-C in group A compared to C. These results have an important significance considering that the present experiment has been conducted on intact rats, and not on a hyperlipidemic murine model. Moreover, HDL-C plays an important role in the epidemiology of coronary heart disease and it is considered to be an independent predictive factor for cardiovascular disease mortality [24]. As LDL is not a major carrier for cholesterol in Wister rats, as it is in humans [25], we did not expect major changes of the plasma LDL-C in this experimental setting.

Our findings are consistent with several human and animal studies. It has been shown that high doses of AAS induce negative effects on plasma lipid profile in athletes, by increasing TG levels and decreasing HDL-C levels up to 70%, mainly HDL2-C subfraction, considered the one that provides maximum anti-atherosclerotic protection [5,26,27]. Unfortunately, most studies conducted in athletes are observational and include subjects with reported intake of other AAS beside DECA, in a scheme that usually combines oral and parenteral AAS. Therefore, it would be difficult to estimate the effect of DECA by itself [8]. The few available human studies that evaluate the independent effect of DECA on lipid profile are contradictory. While some show a detrimental influence of nandrolone on TG and HDL-C fraction [23,28], others find either a mild effect or even a lack of action [29,30]. Animal experiments also reveal contradictory outcomes regarding the effect of DECA on HDL-C, and generally on the whole lipid profile [31,32,33,34]. Data coming from studies assessing DECA influence on lipid profiles are inconclusive due to the small number of experiments and human reports focused on nandrolone use, also due to conflicting results, unvalidated mechanisms, variable baseline lipid profile, differences in study design, including time course, doses of AAS administration, and inhomogeneity of the studied population (healthy athletes, men with HIV, hemodialyzed patients etc, with possible associated comorbidities). The advantage of our experimental setting, using intact rats and not hyperlipidemic ones, is that it better reproduces AAS effects in people who were not previously dyslipidemic: young athletes or amateur bodybuilders abusing of AAS, patients requiring therapeutic AAS administration, or patients with endocrine disorders associating high plasma androgen levels.

A possible mechanism responsible for the DECA-induced plasma TG increase in our study may be high TG hepatic secretion, as TGh levels went down in the androgen-treated group versus controls. We also showed the absence of fatty liver (LW/BW was unchanged in A group vs. C group) and a slightly elevated NEFA (tending to reach statistical significance) in A group vs C group. All these findings, along with the presence of decreased plasma HDL-C cholesterol in A group vs. C group, might be produced by high triglyceride lipase synthesis and activity. AAS abuse was clearly proven to increase the activity of this enzyme (up to 143–232%), which may further be involved in HDL degradation [8,26]. However, there are only a few studies assessing the effects of DECA on liver function [32], and even fewer investigating their influence on hepatic lipid content. A study conducted by Aparicio et al. in rats with normo-cholesterolemic diet and DECA administration, has shown a slightly increased hepatic TG content, an increase of plasma TG and a marked decrease of plasma HDL-C, indicating that DECA may exert important negative effects on lipid profiles in a different experimental setting [35]. Data resulting from a comprehensive study including 180 bodybuilders, has recently demonstrated that AAS abuse could represent a possible new risk factor for “toxicant associated steato-hepatitis” or “toxicant-associated fatty liver disease” development [36].

Regarding the effect of taurine on plasma lipid profile, our research revealed a significant decrease of plasma TG in the mixed treated AT group compared to A group, but not in AT group vs. controls, indicating that taurine may have a potential restorative action centered on serum TG. Taurine did not exert any influence on plasma TG when administered alone, this corresponding to its previously shown effect to induce no or minor changes in intact animals [13,37,38], and important ones in drug or toxic induced pathophysiology [10,39,40]. The neutral effect of taurine on plasma TG may be caused by reduced TG synthesis in the liver, since TGh were significantly low in T group vs. C group. NEFA evaluation came to sustain the fact that taurine seems to diminish hepatic triglyceride lipase activity, as NEFA level showed only nonsignificant variations in T and AT groups versus controls, and a close to statistical significance variation in AT group vs. A group.

Available published data support our findings related to the influence of taurine on plasma TG levels in rats [39,41]. A recent study conducted by Gentile et al. revealed quite similar outcomes, but they used a different protocol of taurine administration. In their study, taurine administered for 4 weeks in rats decreased plasma and liver TG level when added 2% in high sucrose diet, but had no effect when added 2% in standard diet. They have also found that taurine could significantly reduce hepatic lipid accumulation, liver inflammation and injury, concluding that taurine may have the potential to act as a preventive treatment for nonalcoholic fatty liver disease [37]. Human studies have also shown that taurine reduces plasma TG, and has an overall positive effect on plasma lipid profile in overweight or obese individuals, suggesting that it may be useful in cardiovascular disease prevention [14,42].

Regarding the influence of taurine on HDL-C, our results confirm the previous published data, showing its neutral effect when administered alone [38,43]. In contrast, taurine supplementation prevented DECA-induced HDL-C decrease in the mixed treated AT group. This finding is also supported by several other studies, but within a different experimental setting (i.e., high-fat diet administration in rodents) [10,44].

Overall, the influence of taurine on cholesterol metabolism is still a subject of debate, with a considerable amount of contradictory information [10]. Nevertheless, taurine is generally accepted as a dietary supplement that provides a cholesterol lowering effect, especially in exogenous-induced hypercholesterolemia, promoting important regulatory functions in individuals with high dietary cholesterol habits, or with disturbed cholesterol metabolism [10,14].

## 5. Limitations

One limitation could be the lack of an atherosclerosis animal model in this study. However, the hypothesis we wanted to verify was if DECA could produce any changes on lipid profile in intact rats and if taurine could prevent these possible disturbances. AAS doping usually occurs in young normocholesterolemic people, and not all clinical (endocrine) conditions displaying high plasma androgen levels associate dyslipidemia in the initial phase. Another limitation could be the absence of physically trained animals, that otherwise would have better reflected AAS abuse in sporting milieu [22,28,30,31,35]. However, this would have again eluded the endocrine disorders associating elevated concentrations of plasma androgens. The absence of liver histological analysis in order to verify taurine’s beneficial action on DECA-induced damage, is another limitation which resulted from the study design, as tissue specimens haven’t been processed and stored for this type of examination. The small number of animals could also represent a limitation, nevertheless we reached statistical significance. The lipid changes observed in the AT group are obtained after concomitant administration of DECA and taurine, and not sequentially (first DECA, and then taurine in order to test the potential of taurine to reverse DECA negative effects). The experimental design of the present study was set to investigate the potential of taurine to prevent the occurrence of negative effects induced by DECA administration.

## 6. Conclusions

Our study highlights a detrimental influence of DECA in high doses on lipid profile, increasing plasma TG and decreasing plasma HDL-C levels. DECA also decreased TGh and tended to increase NEFA levels in the liver, suggesting an enhancement of hepatic triglyceride lipase synthesis. Taurine supplementation partially prevented the occurrence of these negative effects of steroids on lipids, significantly decreasing the values of TG to control level, and tending to alleviate HDL-C and NEFA variations. On the other hand, taurine seems to exert no influence in control rats. In the light of the clear evidence regarding the proved safety of taurine administration in humans, our study raises the assumption that taurine could be useful in certain circumstances associating chronic high levels of circulating androgens, like endocrine disorders or AAS abuse in athletes. It remains to be verified whether taurine could have a potential health benefit in situations related to AAS misuse, since it represents an important ingredient in boost-energy beverages and it could be a convenient option in individuals not responding to medical AAS-dropout recommendation. Certainly, more data are needed in order to identify taurine as a really useful dietary supplement in counteracting the harmful effects of supraphysiological AAS administration. Recent studies (summarized in ref [45]) highlighted the potential of microRNAs (miRNAs) molecules to become biomarkers of AAS use/abuse and to develop new tools in the anti-doping fight. In this context, further studies are needed to identify specific miRNAs DECA or taurine-dependent, molecules that are able to modulate the expression of key proteins and their transcriptional regulators involved in lipid metabolism. These miRNAs molecules could become potential targets for epigenetic therapies to stop or reverse damages induced by AAS consumption. Furthermore, the association of aerobic / anaerobic activity with the combined use of DECA and taurine could simulate the real circumstances of steroid abuse.

## Figures and Tables

**Table 1:** Values of triglycerides (TG), total cholesterol (TC), low density lipoprotein cholesterol (LDL-C), high density lipoprotein cholesterol (HDL-C), liver triglycerides (TGh), liver non-esterified fatty acids (NEFA), and the ratio of the liver weight relative to the body weight (LW/BW) in rats treated with vehicle (C Group), nandrolone decanoate (A Group), taurine (T Group) and their combination (AT Group);.

Variable	C group	A Group	T Group	AT Group	*p* ANOVA
TG (mg/dL)	56.33 ± 10.74	85.15 ± 21.44	63.22 ± 12.0	60.24 ± 23.26	0.01
TC (mg/dL)	58.83 ± 5.50	57.54 ± 8.46	60.44 ± 8.23	58.33 ± 10.51	0.91
LDL-C (mg/dL)	20.04 ± 3.49	20.67 ± 2.90	23.53 ± 3.06	21.10 ± 2.11	0.11
HDL-C (mg/dL)	46.00 ± 6.64	33.90 ± 4.67	44.33 ± 10.69	37.33 ± 7.68	0.01
TG_h_ (µg/mg protein)	657.022 ± 102.45	536.61 ± 23.33	506.50 ± 111.09	470.04 ± 43.48	*p* < 0.001
NEFA (µmol/mg protein)	11.05 ± 2.35	15.66 ± 7.27	13.78 ± 4.01	10.19 ± 1.17	0.06
LW/BW (g/g)	0.036 ± 0.003	0.038 ± 0.002	0.037 ± 0.002	0.036 ± 0.002	0.21

*p* ANOVA represents the level of statistical significance using one-way analysis of variance.
